# The auditory efferent system in mosquitoes

**DOI:** 10.3389/fcell.2023.1123738

**Published:** 2023-02-27

**Authors:** YuMin M. Loh, Matthew P. Su, David A. Ellis, Marta Andrés

**Affiliations:** ^1^ Graduate School of Science, Nagoya University, Nagoya, Aichi, Japan; ^2^ Institute for Advanced Research, Nagoya University, Nagoya, Aichi, Japan; ^3^ UCL Ear Institute, University College London, London, United Kingdom; ^4^ The Francis Crick Institute, London, United Kingdom

**Keywords:** hearing, anopheline and culicine mosquitoes, auditory efferent system, neurotransmitters, Johnston’s organ, octopamine, serotonin, GABA

## Abstract

Whilst acoustic communication forms an integral component of the mating behavior of many insect species, it is particularly crucial for disease-transmitting mosquitoes; swarming males rely on hearing the faint sounds of flying females for courtship initiation. That males can hear females within the din of a swarm is testament to their fabulous auditory systems. Mosquito hearing is highly frequency-selective, remarkably sensitive and, most strikingly, supported by an elaborate system of auditory efferent neurons that modulate the auditory function - the only documented example amongst insects. Peripheral release of octopamine, serotonin and GABA appears to differentially modulate hearing across major disease-carrying mosquito species, with receptors from other neurotransmitter families also identified in their ears. Because mosquito mating relies on hearing the flight tones of mating partners, the auditory efferent system offers new potential targets for mosquito control. It also represents a unique insect model for studying auditory efferent networks. Here we review current knowledge of the mosquito auditory efferent system, briefly compare it with its counterparts in other species and highlight future research directions to unravel its contribution to mosquito auditory perception.

## Introduction

Mosquitoes are endowed with some of the most complex ears in the animal kingdom. Despite their small size (∼200 µm in diameter), male mosquito ears contain around 15,000 auditory neurons (Boo and Richards, 1975a), which approximates the number of hair cells in the human cochlea. Like the tone of their wingbeats, the mosquito ear and its associated acoustic information-processing brain regions (Li et al., 2022) show high sexual dimorphism, likely reflecting the differential contributions of hearing towards the reproductive biology of male and female mosquitoes. Courtship is initiated by males using their hearing organs to detect female flight tones (Clements, 1999). For most mosquito species, this courtship ritual takes place in male-dominated aerial swarms, in which up to a thousand mosquitoes can aggregate simultaneously (Diabate and Tripet, 2015; Sawadogo et al., 2017). As such, the swarm is a challenging sensory environment where males detect the faint flight tones of their mating partners in a noisy context. This demanding sensory ecology has likely acted as an evolutionary driving force to shape the mosquito auditory system, which requires both frequency sensitivity and selectivity.

Perhaps the most striking signature of the intricacy of the mosquito hearing system at both the anatomical and functional levels is their auditory efferent system, the only documented example amongst insects (Andrés et al., 2016). From a functional perspective, neurons are classified as afferent or efferent depending on the direction of information flow. Afferent neurons carry information to the central nervous system; efferent neurons carry information away from the central nervous system to the periphery. Sensory efferent systems are therefore descending pathways from the central nervous system to sensory organs that modulate their function (Ryugo et al., 2010). The mosquito auditory efferent system in mosquitoes, which contains a variety of neurotransmitters, multiple distinct sites of release and different functional effects, matches the complexity of their vertebrate counterparts (Andrés et al., 2016; Su et al., 2018; Georgiades et al., 2022).

In humans, efferent activity in the ear has been related to the extraction of relevant sounds in noisy environments (Ryugo et al., 2010). Likewise in mosquitoes, the selective amplification of relevant sounds in a swarm seems to be essential for detecting the flight tones of a mating partner (Su et al., 2018). Considering the highly transient and dynamic nature of swarms, neuromodulation of the male ear *via* an extensive efferent network would confer them with a layer of auditory plasticity necessary for the swift modulations of their auditory function to meet their hearing needs.

Given that mosquitoes act as significant vectors of disease, understanding the fundamental principles of their auditory efferent system is not only relevant from a basic sensory biology perspective, but also pertinent in the context of identifying suitable molecular targets that could inform the design of novel mosquito control interventions. Moreover, because of its accessibility and the potential for common underlying mechanisms, the mosquito ear could also be a model to understand how efferent control modulates auditory function across species. In this review, we shall discuss the current knowledge of mosquito auditory efferent systems, compare and contrast their anatomical and functional features with auditory efferent systems in other species, and outline the steps necessary to further elucidate the fundamental components which underlie this unique system.

## Fundamental anatomy and function of mosquito ears

**FIGURE 1:**
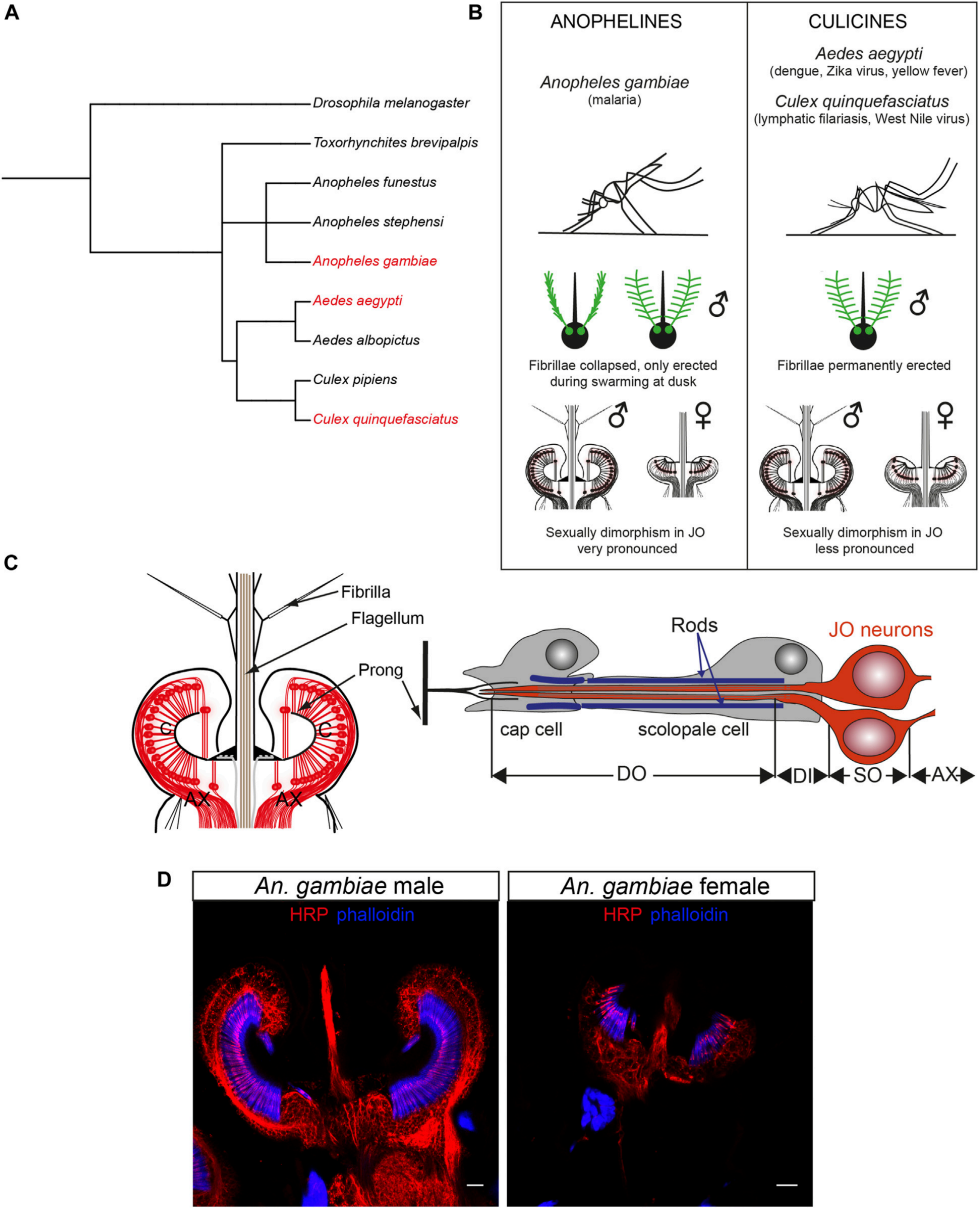
Mosquito auditory systems. **(A)** Phylogenetic tree of mosquito evolution including the three medically-relevant species reviewed in this manuscript (*Ae. aegypti, An. gambiae, Cx. quinquefasciatus*), other mosquito species and the model organism *D. melanogaster*. Made with PhyloT. **(B)** Summary of the main differences between anopheline (*An. gambiae*) and culicine (*Ae. aegypti, Cx. quinquefasciatus*) mosquitoes relevant for hearing research. **(C)** Schematic of the mosquito JO (left) and a single type A scolopidium (right) depicting two auditory neurons, supporting cap and scolopale cell. Modified from (Andrés et al., 2016). **(D)** Sexual dimorphism in male (left) and female (right) *An.* gambiae JOs stained with neuronal marker anti-HRP (red) and phalloidin (F-actin marker, blue) that binds to actin rods in scolopale cells surrounding the auditory cilia. The female ear is smaller and less complex than the male counterpart. Scale bar: 10 µm. AX: afferent axons; C: auditory cilia; DI: dendritic inner segment; DO: ciliated dendritic outer segment; JO: Johnston’s organ; SO: somata.

Some mosquito species act as disease vectors, causing great suffering across human populations. Because of their public health relevance, research on mosquito biology has been mostly focused on a few mosquito species, including the malaria mosquito *Anopheles gambiae* (*An. gambiae*), the dengue mosquito *Aedes aegypti* (*Ae. aegypti*), and the southern house mosquito *Culex quinquefasciatus* (*Cx. quinquefasciatus*), vector of lymphatic filariasis and other arboviruses (Figures 1A, B). Despite the fact that diversification between anopheline (*An. gambiae*) and culicine (*Ae. aegypti* and *Cx. quinquefasciatus*) mosquitoes occurred around 182 million years ago (da Silva et al., 2020), the anatomy of their ears remains broadly conserved.

Mosquitoes hear *via* antennal ears comprised of a sound receiver (or flagellum), and the actual auditory organ, the Johnston’s organ (JO), enclosed in the second antennal segment (Figure 1C) (Göpfert et al., 1999; Göpfert and Robert, 2000). In contrast to vertebrate ears, which detect changes in pressure waves emitted by a sound source, mosquito ears detect the particle velocity component of a sound field, or sound-induced air particle vibrations. As such, the light flagellum is set in motion by friction with the air particles around it that vibrate when, for example, a nearby mosquito beats its wings (Albert and Kozlov, 2016). The flagellum is covered by small hairs (fibrillae) that likely aid in increasing its sensitivity to sound (Göpfert et al., 1999); male hairs are far longer and more numerous than conspecific female fibrillae, and, in some mosquito species, such as the malaria mosquito *An. gambiae*, they only become erect at a specific time of day when mosquitoes swarm (Pennetier et al., 2010). Nanometre-scale flagellar vibrations are transmitted through the flagellar base, the basal plate, to cuticular couplings called prongs and ultimately to ciliated auditory neurons in the JO that transduce and convert the mechanical stimulations into electrical signals that propagate to the brain (Göpfert and Robert, 2000).

A series of elegant studies initiated in the 1970s revealed the general anatomy of the mosquito JO (Boo and Richards, 1975a; 1975b; Boo, 1980), which is highly sexually dimorphic (Figures 1B, D). The male JO typically contains around 15,000 neurons (as many as hair cells in the human cochlea), which is about twice the number of JO neurons in females across different mosquito species (∼7000 neurons). By comparison, the JO of the predominant insect model *Drosophila melanogaster* (*D. melanogaster*) comprises only around 500 sensory neurons (Göpfert and Albert, 2006; Kamikouchi et al., 2006).

Sexual dimorphism is also present at the auditory behavioural level. Males are attracted to the flight tones of females and use the sounds emitted by flying females in the swarm to acoustically locate them for mating (Roth, 1948; Belton, 1994). By contrast, whilst probing auditory responses in female mosquitoes is possible (Bartlett-Healy et al., 2008), the lack of clearly defined female acoustic behaviours makes the significance of female mosquito audition unclear.

The auditory neurons are both ciliated and bipolar, and are assembled in groups of 2–3 that, together with supporting cells, form functional mechanosensory units, the scolopidia (Figure 1C). Scolopidia have been traditionally divided into different groups depending on their morphology and location. Type A scolopidia harbour two auditory neurons and make up 97% of all JO scolopidia. Type B scolopidia contain three auditory neurons, are located distally in the pedicel and comprise almost all of the remaining 3% of the scolopidia. Both types of scolopidia are amphinematic (i.e., the sensory cilia is surrounded by a tubular electro-dense that connects to the cuticle) and are involved in hearing, although the functional differences between both types remain elusive (Field and Matheson, 1998; Yack, 2004). The JO also contains two type C and a single type D scolopidia (absent in females of most mosquito species), which contain two sensory neurons each and differ from type A and B in that the supporting cap cell anchors the sensory neurons to the epidermis under the basal plate (mononematic scolopidia) (Field and Matheson, 1998; Yack, 2004). Types C and D are likely proprioceptive, rather than auditory, in nature.

The auditory afferent axons have been proposed to project to the Johnston´s organ centre (JOC), a multilobed structure within the antennal lobe in the mosquito deuterocerebrum (Ignell et al., 2005). However, some recent research suggest instead that they project to the antennal motor and mechanosensory center (AMMC), as previously described in *Drosophila* (Kamikouchi et al., 2006; Xu et al., 2022). Non-auditory flagellar neurons (i.e., the olfactory/chemosensory neurons within the flagellum itself) project to a distinct section of the antennal lobe (Shankar and McMeniman, 2020).

We are only now starting to understand how the neuronal complexity of the mosquito JO mediates sound perception. *Drosophila* JO neurons, which are 30 times less abundant than in mosquitoes (Eberl and Boekhoff-Falk, 2007), were previously divided into five anatomical subgroups, A-E, that project to different regions of the AMMC (Matsuo et al., 2014) and detect different type of mechanosensory stimuli (Kamikouchi et al., 2006, 2009; Yorozu et al., 2009; Matsuo et al., 2014). Even for this relatively low number of auditory neurons, new sub-groupings are still being identified, with a sixth subgroup (referred to as JO-F) being recently described (Hampel et al., 2020). We do not know if these functional subgroups exist in mosquitoes. Lapshin and Vorontsov have reported functional specialization across scolopidia that differ in their frequency tuning (Lapshin and Vorontsov, 2013, 2019). In *Culex pipiens* mosquitoes, eight groups of auditory neurons have been reported, a majority of them being sensitive to 190–270 Hz (Lapshin and Vorontsov, 2017), a frequency range that nicely encompasses the quadratic and cubic distortion product frequencies that are generated by the non-linear mixing of male and female flight tones and have been suggested to mediate the female detection by the male (Somers et al., 2022). Whether scolopidia in the mosquito JO are tonotopically arranged is still unknown.

Although, anatomically, vertebrate and mosquito ears might appear completely different, functionally they share fundamental features that aid in boosting sound detection, namely, active frequency tuning, mechanical amplification of faint sounds and potentially a compressed dynamic range to increase the ear response to different sound intensities (Göpfert and Robert, 2001; Albert and Kozlov, 2016; Warren and Nowotny, 2021). These properties shared by vertebrates and mosquitoes can be partially explained by the motor function of auditory cells which, apart from playing sensory roles, also contribute to the active amplification of mechanical stimuli by injecting mechanical energy (Fettiplace and Hackney, 2006; Karak et al., 2015). In mammals, outer hair cell (OHC) electromotility mediated by the voltage-dependent motor protein Prestin (Zheng et al., 2000; Oliver et al., 2001; Warren and Nowotny, 2021) and hair-bundle electromotility driven by calcium-currents are the suggested mechanisms that explain the cochlear amplifier [or the active amplification of low-level and compression of high-level basilar membrane displacements caused by sounds of different intensity (Ashmore et al., 2010)].

In insect antennal ears, such as the mosquito JO, mechanical amplification appears to be independent of Prestin (Kamikouchi et al., 2010), but rather mediated by the motile properties of ciliated auditory neurons and the force generated by transduction channels coupled to adaptation motors and gating springs (Göpfert and Robert, 2001, 2003; Göpfert and Albert, 2006; Mhatre, 2015). A signature of active mechanical amplification of sound in both vertebrate and mosquito ears is the occurrence of spontaneous, frequency-specific vibrations of the ear in the absence of sound, so-called spontaneous otoacoustic emissions (SOAEs) in vertebrates (Dallos, 1992; Hudspeth, 1997) and self-sustained oscillations (SSOs) in mosquitoes (Göpfert and Robert, 2001; Su et al., 2018). Mosquito SSOs appear spontaneously only in males as large, mono-frequent vibrations of around 350 Hz, though they can be induced in females *via* injection of dimethyl sulfoxide (DMSO) (Su et al., 2018). Although the biological significance of male-specific SSOs is yet to be fully elucidated, they have been suggested to act as built-in signal amplifiers of female flight tones, as the frequency range of both significantly coincides (Warren et al., 2009; Pennetier et al., 2010; Su et al., 2018).

## Anatomy of the mosquito auditory efferent system

Efferent input was long thought to be a unique feature of vertebrate hearing. Functional studies into potential efferent systems in *D. melanogaster*, the core insect hearing model, found no apparent dependence of auditory neuron function on efferent modulation. Parallel immunohistochemistry studies likewise concluded a lack of evidence of peripheral synapses within the *Drosophila* JO (Kamikouchi et al., 2010). Though this exclusion of an efferent modulation of JO neuronal activities has been since slightly modified (see below), presynaptic terminals, the existence of which is indicative of the release of neurochemicals *via* efferent innervation, are essentially lacking within the *Drosophila* JOs.

**FIGURE 2:**
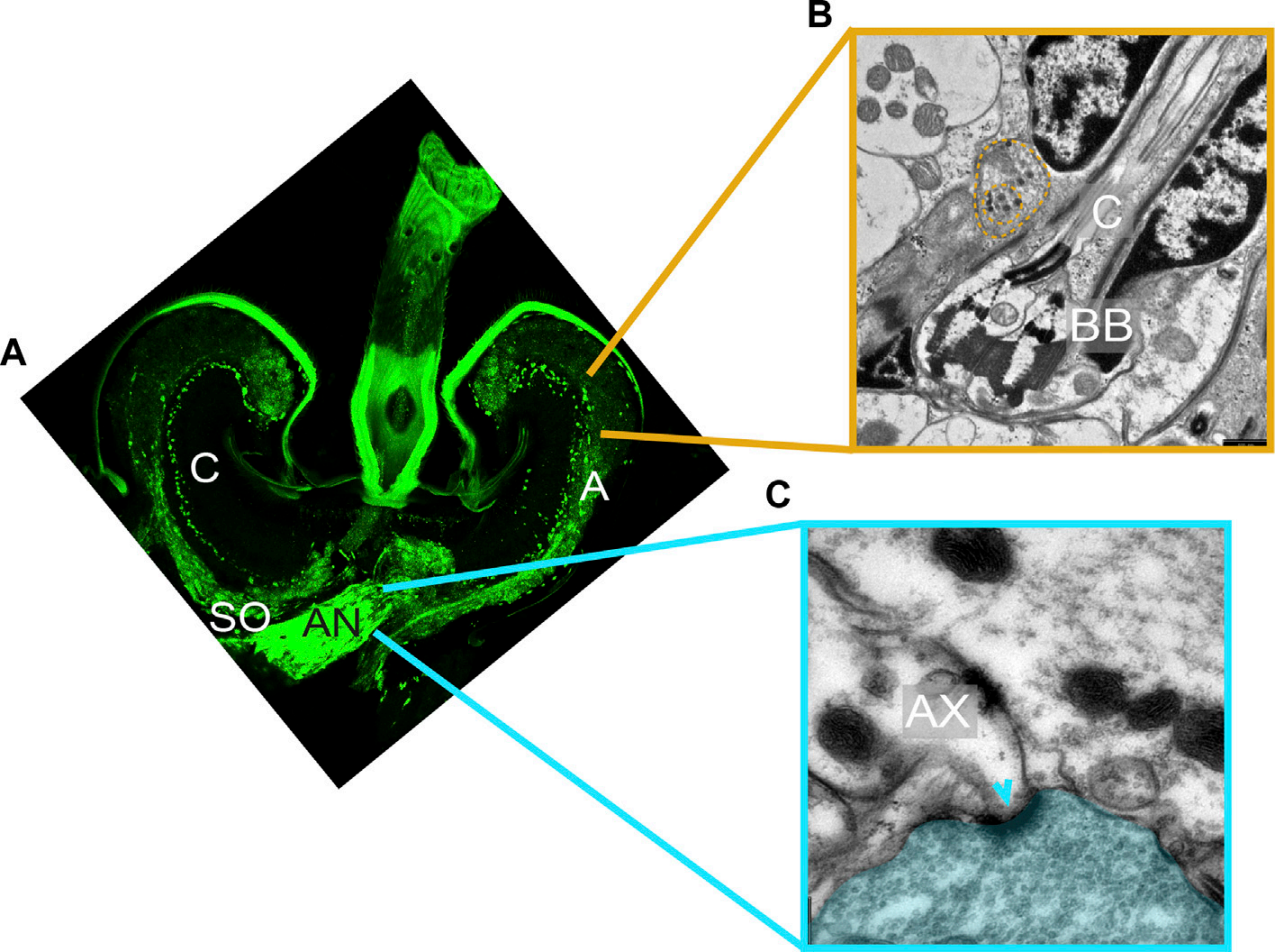
Ultrastructure of auditory efferent terminals. **(A)** Immunostaining of a male JO labelling the presynaptic protein synapsin (mAb 3C11, anti-synapsin, green) suggesting the presence of presynaptic terminals within the male JO. Modified from Andrés et al. (2016). **(B,C)** Transmission electron microscope images corresponding to distinct sections of a male *Cx. quinquefasciatus* JO. **(B)** Ciliary base region, showing a terminal filled with DCVs (yellow dashed line) located in close proximity to the neuronal membrane around basal bodies. **(C)** Auditory nerve region depicting two axons and an adjacent efferent fibre filled with SVs (blue area). A clear synaptic specialization in the postsynaptic axon can be visualized (arrowhead). AN: antennal nerve; AX: afferent axons; BB: basal body; C: auditory cilia; SO: somata.

The first evidence for the presence of auditory efferents in the mosquito ear came from immunohistochemistry in *Cx. quinquefasciatus* (Andrés et al., 2016, 2020). Labelling with the presynaptic markers 3C11 (anti-synapsin) and nc46 (anti-SAP46) revealed a complex staining pattern in multiple sites of the mosquito JO including the base of the auditory cilia, the auditory neuron somata and axons, the area underneath the basal plate, and along the auditory nerve (Figure 2A). These findings supported the presence of presynaptic sites within the mosquito JO. Retrograde tracing of the efferent fibers and Golgi stainings of the mosquito brain assigned the origin of some of these neurons to an area located in the lateral protocerebrum adjacent to the optic lobes, providing evidence of its efferent nature (Andrés et al., 2016).

Ultrastructural characterization of the efferent terminals in males using transmission electron microscopy (TEM) showed fundamental differences between presynaptic terminals innervating the auditory nerve outside of the JO, and the rest of the terminals identified within the JO (Figures 2B, C). Presynaptic terminals innervating the axons in the auditory nerve were filled with small clear synaptic vesicles (SVs) and the corresponding postsynaptic structures showed clear synaptic specializations (Figure 2C). The abundance of synaptic sites with presynaptic terminals filled with SVs suggests an intrinsic role of these peripheral synapses in modulating the auditory nerve function (Edwards, 1998).

By contrast, presynaptic terminals identified along the ciliary base, somata and axons within the JO do not present postsynaptic specializations in close vicinity, despite being labelled by the presynaptic markers 3C11 and nc46 (Yu et al., 2021), suggesting volume transmission mechanisms of chemical communication (Fuxe et al., 2010; Liu et al., 2021). Instead, presynaptic terminals in these areas are mostly filled with large dense-core vesicles (DCVs), which given the chemical nature of DCVs, could potentially be comprised of biogenic amines and neuropeptides. Presynaptic terminals were observed in the vicinity of all inner dendritic segments (Figure 2B), suggesting that all type A and B scolopidia receive efferent input. However, because of the absence of clear postsynaptic structures, it was not possible to decipher the target cells of these presynaptic terminals at the ciliary base, as both auditory neuron and scolopale cell membranes were in close proximity. The location of terminals along the somata and axons within the JO was less stereotyped, probably due to a less organized cellular arrangement of these JO regions.

**FIGURE 3:**
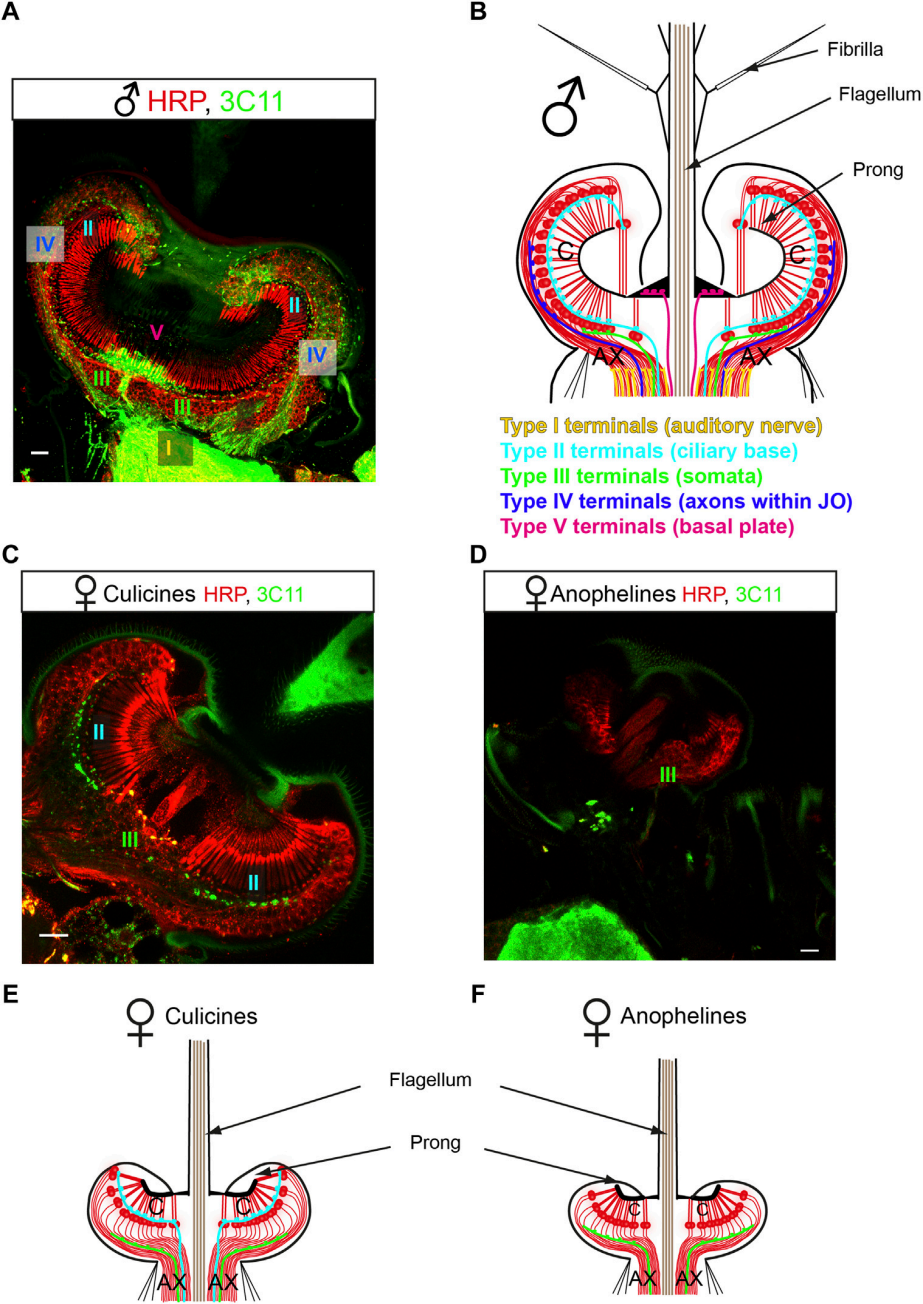
New nomenclature proposed for the mosquito auditory efferent system. **(A,C,D)** Efferent terminals innervate the male and female mosquito JOs. JO horizontal sections stained with a presynaptic marker (mAb 3C11, anti-synapsin, green) and counterstained with a neuronal marker (anti-HRP, red). **(A)** Male mosquitoes (here a *Cx. quinquefasciatus* male JO) present an extensive efferent innervation that targets the auditory nerve (type I terminals), the ciliary basis (type II terminals), the somata (type III terminals), the axons within the JO (type IV terminals) and the basal plate (type V terminals). **(B)** Schematic figure summarising the distribution of efferent presynaptic terminals in the male JO. This pattern is consistent in males across anopheline and culicine mosquitoes. **(C,D)** Female mosquitoes present a reduced efferent system compared to males, but differences are clear between culicines **(C)** and anopheline **(D)** mosquitoes. **(C)** Culicine female (here a *Cx. quinquefasciatus* female JO) present type II and III terminals, innervating the auditory neuron cilia and somata, respectively. **(D)** *An. gambiae* females present a drastically reduced efferent innervation, with some sparse type III terminals observed across the neuronal cell bodies. **(E,F)** Schematics summarizing the distribution of the auditory efferent terminals in females. Culicine females **(E)** present a more extensive auditory efferent pattern compared to anopheline females **(F)**. Modified from (Andrés et al., 2016) and (Su et al., 2018). AX: afferent axons; C: auditory cilia. Scale bar: 10 μm.

Due to the complexity of the efferent pattern, we propose in this article a new terminology for the efferent terminals innervating the mosquito JO based on their localization and ultrastructural features. We define five different efferent terminal types (Figure 3).

1) Type I terminals innervate the auditory nerve outside the JO and are ultrastructurally characterized by being filled with SVs and presenting clear synaptic specializations on the postsynaptic site, hinting at a subnanometer scale, close-range mode of intercellular chemical communication by synaptic transmission;

Types II-V are filled with DCVs and do not present synaptic specializations in the postsynaptic site, suggestive of a volume transmission mode of chemical release. Types II-V are classified depending on their location as:

2) Type II terminals that innervate the inner dendritic segments of auditory neurons close to the ciliary basal bodies and ciliary rootlets;
3) Type III terminals that are scattered across neuronal somata;
4) Type IV terminals that innervate the auditory neuron axons within the JO before joining the auditory nerve;
5) Type V terminals that innervate the region underneath the basal plate.

A pioneering study compared the auditory efferent innervation in *An. gambiae, Ae. aegypti and Cx. quinquefasciatus* (Su et al., 2018). Across males, despite some minor species-specific differences in terminal distribution, all five different terminal types were present and the general organization was well-conserved across mosquito species despite their evolutionary divergence around 180 million years ago (Figures 3A, B). This evolutionary conservation suggests an inherent role of the efferent system in mosquito auditory function. This work also showed a strong sexual dimorphism in efferent innervation and greater variability in the efferent patterns across females of these mosquito species (Figures 3C–F) (Su et al., 2018). While *Ae. aegypti* and *Cx. quinquefasciatus* females presented some type II and III terminals, female *An. gambiae* had very few terminals (probably of III) and the extent of the system was dramatically reduced (Su et al., 2018).

## Pharmacology of the auditory efferent system in mosquitoes

**FIGURE 4:**
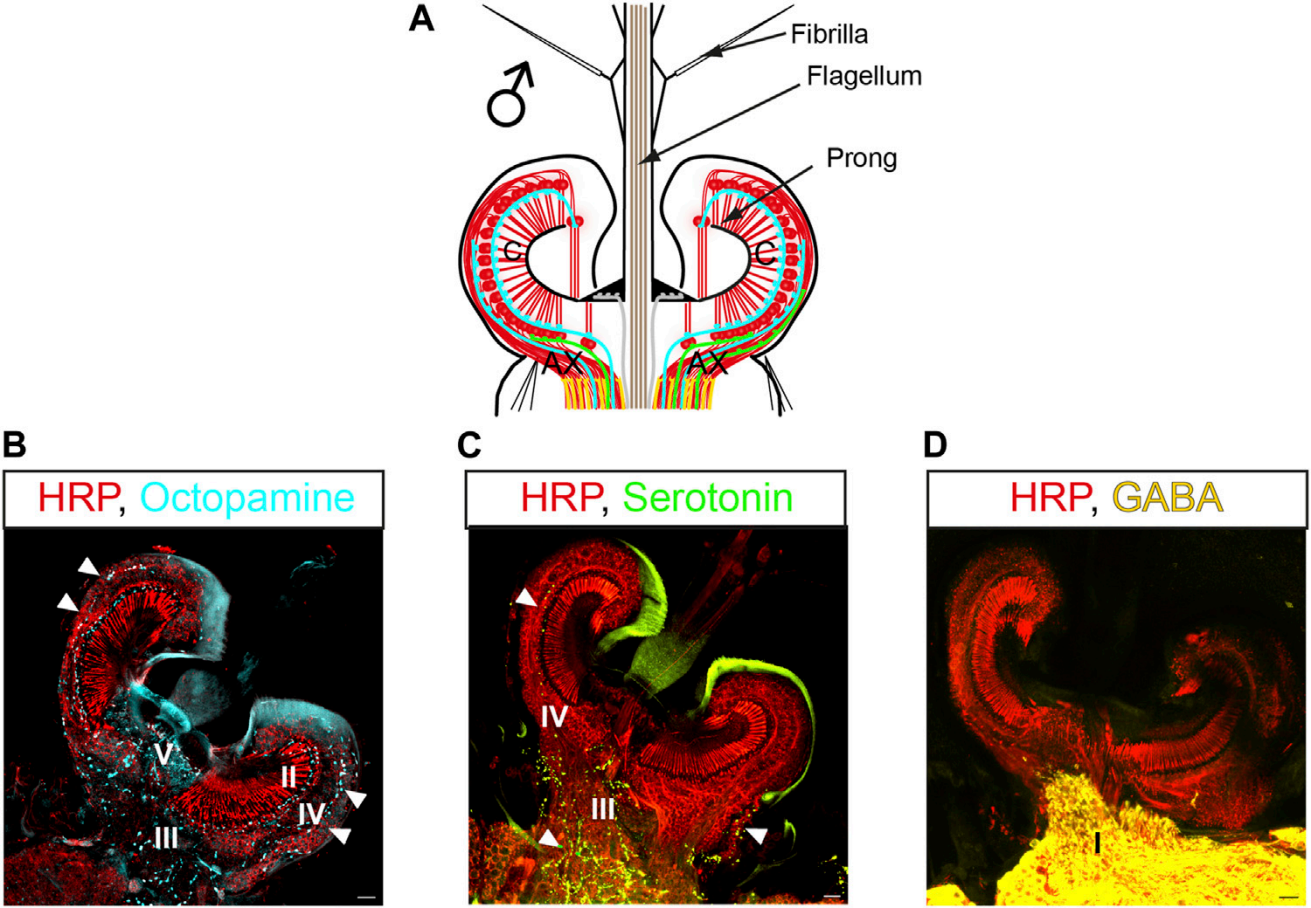
Pharmacology of the auditory efferent system in mosquitoes. **(A)** Schematic of a male JO and the efferent presynaptic terminals corresponding to the color coding in **(B–D)**; efferent terminals shown as lines correspond to blue: octopamine, green: serotonin, yellow: GABA. **(B–D)** JO horizontal sections stained with antibodies recognizing different neurotransmitters and counterstained with a neuronal marker (anti-HRP, red), **(B)** anti-octopamine (light blue, arrowheads); **(C)** anti-serotonin (green, arrowheads) and **(D)** anti-GAD (yellow, enzyme required for GABA synthesis). Letters in pictures represent the type of efferent terminals that release each neurotransmitter. Modified from (Andrés et al., 2016). AX: afferent axons; C: auditory cilia. Scale bar: 10 µm.

Immunohistochemical characterizations in *Cx. quinquefasciatus* and *Ae. aegypti* have revealed which neurotransmitters are being released at the different terminal types (Figure 4) (Andrés et al., 2016; Xu et al., 2022). The biogenic amines octopamine and serotonin are released from terminals within the JO (terminals II-V, Figures 4B, C), and the inhibitory neurotransmitter γ-aminobutyric acid (GABA) is released from type I terminals in the auditory nerve (Figure 4D). This agrees with the TEM observations previously described, as biogenic amines are stored in DCVs while amino-acid neurotransmitters are stored in SVs (Stocker et al., 2018).

In *Cx. quinquefasciatus* males, octopamine is released from type II, III, IV, and V terminals (Andrés et al., 2016). Serotonin efferent patterns in males are highly conserved between *Cx. quinquefasciatus* (Andrés et al., 2016) and *Ae. aegypti* (Xu et al., 2022), with serotonin being released from III and IV terminals. Although anatomical confirmation is lacking in *An. gambiae* males, a similar neurotransmitter distribution is expected due to similarities in their efferent innervation pattern.

A recent paper has reported the expression of a broad range of receptors from different neurotransmitter families in the ear of male *An. gambiae* mosquitoes (Georgiades et al., 2022), including not only octopamine, serotonin and GABA, but also other classical neurotransmitters such as acetylcholine (ACh) and glutamate. It is therefore plausible that ACh and glutamate are also released from efferent terminals in the mosquito JO. Further investigation is needed to understand the full chemical and neuroanatomical nature of the mosquito auditory efferent system.

Interestingly, the same study also found expression of multiple neurotransmitter receptors in the JO of female *An. gambiae* mosquitoes (Georgiades et al., 2022), despite scarce labelling with presynaptic markers (Su et al., 2018). Due to the smaller size of the female JO compared to the male, it is plausible that volume transmission mechanisms suffice to mediate the diffusion of neurotransmitters from the brain to the receptors in the JO (Liu et al., 2021; Guidolin et al., 2022). Some neurotransmitters might also reach the JO *via* the haemolymph as neurohormones. Furthermore, it is also possible that some of the neurotransmitter receptors localize to the auditory nerve connecting the JO to the brain. Dissections for RNA-Seq involve removing whole pedicel from the head, making it is plausible that the dissected material might contain non-JO axonal sections (Georgiades et al., 2022), potentially explaining the discrepancy between transcriptomic and immunohistochemistry data.

Relevant to this, a recent RNA-Seq analysis of *Drosophila* JOs, previously reported to lack any apparent efferent signatures (Kamikouchi et al., 2010), also found significant levels of gene expression for a broad range of neurotransmitter receptors (Keder et al., 2020). The authors hypothesized that the explanation for an apparent lack of synaptic activity within the *Drosophila* JO but an enriched receptor expression profile could be that these receptors are located in the axonal membrane of JO auditory nerve, closer to the AMMC in the brain. A recent electron-microscopy-based work led by Kim and others supports this explanation (Kim et al., 2020), with their images showing the auditory nerve outside of the JO as possessing postsynaptic sites, hinting at a potential role of these innervations in modulating the auditory electrical signal en route to AMMC. Studying the localisation of neurotransmitter receptors using antibodies (Gregor et al., 2022) or other genetic approaches will be important to validate *omics* data and to understand the mechanisms of chemical transmission that mediate the neuromodulation of the JO physiology in mosquitoes.

## Function of the auditory efferent system in mosquitoes

Mosquito auditory function has been studied using laser Doppler vibrometry (LDV) to record mechanical responses of the flagellum (sound receiver). Corresponding electrical responses of the nerve have been measured using electrophysiology. Analytical pipelines include measuring the spontaneous vibrations of the flagellum in the absence of sound (free fluctuations), followed by mechanical stimulation exposure. Different parameters such as the flagellar mechanical state, mechanical and electrical frequency tuning (including best frequency and tuning sharpness), sensitivity and stiffness of the system can be extracted from these measurements (Andrés et al., 2016; Su et al., 2018; Georgiades et al., 2022). Analysing the effects of individual neurotransmitters on these parameters is important to understand how the efferent system influences the mosquito auditory perception and future functional studies should take them into account (Table 1). It is important to consider that emerging properties of the system might be challenging to study given the limitations of the experimental setup. Examining potential circadian time-dependent changes in the effects of the efferent neurotransmitters is also necessary, as mosquito auditory physiology is influenced by circadian time (Georgiades et al., 2022).

**TABLE 1:** Relevant auditory parameters to be analysed for auditory efferent effects.

Stimulation type	Recording type	Parameters	References
Unstimulated, free fluctuations	LDV	Flagellar mechanical state, best frequency, tuning sharpness, amplitude, power gain	Su et al. (2018); Georgiades et al. (2022)
White noise (*via* loudspeaker playback or electrostatic actuation)	LDV	Best frequency, displacement gain	Andrés et al. (2016); Xu et al. (2022)
Force-steps (*via* electrostatic actuation)	LDV, electrophysiology	Flagellar stiffness, mechanical and electrical sensitivity	Su et al. (2018); Georgiades et al. (2022)
Frequency-modulated sweeps (*via* loudspeaker playback or electrostatic actuation)	LDV, electrophysiology	Mechanical and electrical frequency tuning, maximum flagellar sensitivity, flagellar fluctuation power	Andrés et al. (2016); Georgiades et al. (2022)

### Global effects

Given the lack of neurotransmitter receptor antibodies for mosquitoes, as well as a paucity of genetic tools, it has been challenging to identify the nature and expression pattern of neurotransmitter receptors located within the JO. However, recent advancements in mosquito genome engineering using CRISPR-Cas9, such as the generation of promoter-specific QF driver lines and QUAS-fluorescent reporter marker line, have opened up new avenues to finally start characterising the sensory neurons determining mosquito behaviors (Coutinho-Abreu and Akbari, 2022; Herre et al., 2022; Konopka et al., 2022).

Although antibodies and genetic resources are not yet widely available, researchers have already started profiling the functional role of the auditory efferent system by interfering with the function of all receptors within the JO *via* injection of either tetrodotoxin (TTX) or tetanus toxin (TeNT) to disrupt all afferent/efferent signalling pathways, thus acting as a palimpsest of more targeted *Drosophila* experiments driving TTX specifically in auditory neurons (Kamikouchi et al., 2010). Injection of either TTX or TeNT resulted in males exhibiting SSOs, whilst females showed essentially no change when compared to either pre-injection status or control injections with a physiological Ringer solution (Su et al., 2018). That severing efferent signalling led to SSO initiation suggests that some component of this efferent system controls SSO onset; however, it remains unclear exactly which neurochemicals are responsible for driving this phenomenon.

### Octopamine and tyramine

The two biogenic amines octopamine and tyramine are invertebrate-specific counterparts of the vertebrate adrenergic neurotransmitters adrenaline and noradrenaline (Roeder, 2005). Invertebrate and vertebrate systems share many similarities regarding neurotransmitter biosynthesis pathways, pharmacology and signalling *via* G-protein-coupled receptors (GPCRs) (Farooqui, 2007). The octopaminergic/tyraminergic system of insects controls a plethora of vital physiological processes and behaviours, including locomotion, egg laying, feeding, olfaction, sleep and mating (Linn et al., 1996; Flecke and Stengl, 2009; Ma et al., 2015; Schendzielorz et al., 2015; Zhukovskaya and Polyanovsky, 2017).

Putative octopamine release sites were found in type II, III, IV and V terminals of male *Cx. quinquefasciatus* mosquitoes, as described above (Figures 4A, B) (Andrés et al., 2016). The functional significance of this octopaminergic innervation was tested using a pharmacological approach. Thoracic injections of octopamine or the octopamine receptor agonist, clonidine, were performed, and effects in the flagellar mechanics were measured by LDV. Both compounds affected flagellar mechanics and shifted mechanical frequency tuning to higher values. They increased flagellar sensitivity to sound and boosted the fluctuation power of male ear vibrations, indicating enhanced JO neuron motility and mechanical amplification (Table 2). Further administration of the octopamine receptor antagonist, phentolamine, induced a near complete reversion of the flagellar mechanics to its pre-activation state, demonstrating the specificity of the octopaminergic effect on (male) hearing function.

**TABLE 2:** Known effects of neurotransmitters on male mosquito hearing function.

Neurotransmitter type	Mosquito species tested	JO IHC	Neurotransmitter release sites (presynaptic terminals)	Receptors identified in JO (*via* RNA-Seq or RT-qPCR)	Effect of neurotransmitter exposure on hearing function	References
Octopamine	*Cx. quinquefasciatus*/*An. gambiae*	Yes	II, III, IV, V	Yes	Increase in mechanical tuning frequency and flagellar stiffness. SSO frequency and amplitude modulation	Andrés et al. (2016); Georgiades et al. (2022)
Serotonin	*Cx. quinquefasciatus*/*An. gambiae*/*Ae. aegypti*	Yes	II, III	Yes	Increase in mechanical tuning frequency	Andrés et al. (2016); Georgiades et al. (2022); Xu et al. (2022)
GABA	*Cx. quinquefasciatus*/*An. gambiae*	Yes	I	Yes	Increase in mechanical tuning frequency/change in nerve response	Andrés et al. (2016); Georgiades et al. (2022)
Acetylcholine	*An. gambiae*	No	Unknown	Yes	Unknown	Georgiades et al. (2022)
Glutamate	*An. gambiae*/*Cx. quinquefasciatus*	No	Unknown	Yes	Unknown	Andrés et al. (2016); Georgiades et al. (2022)

**FIGURE 5:**
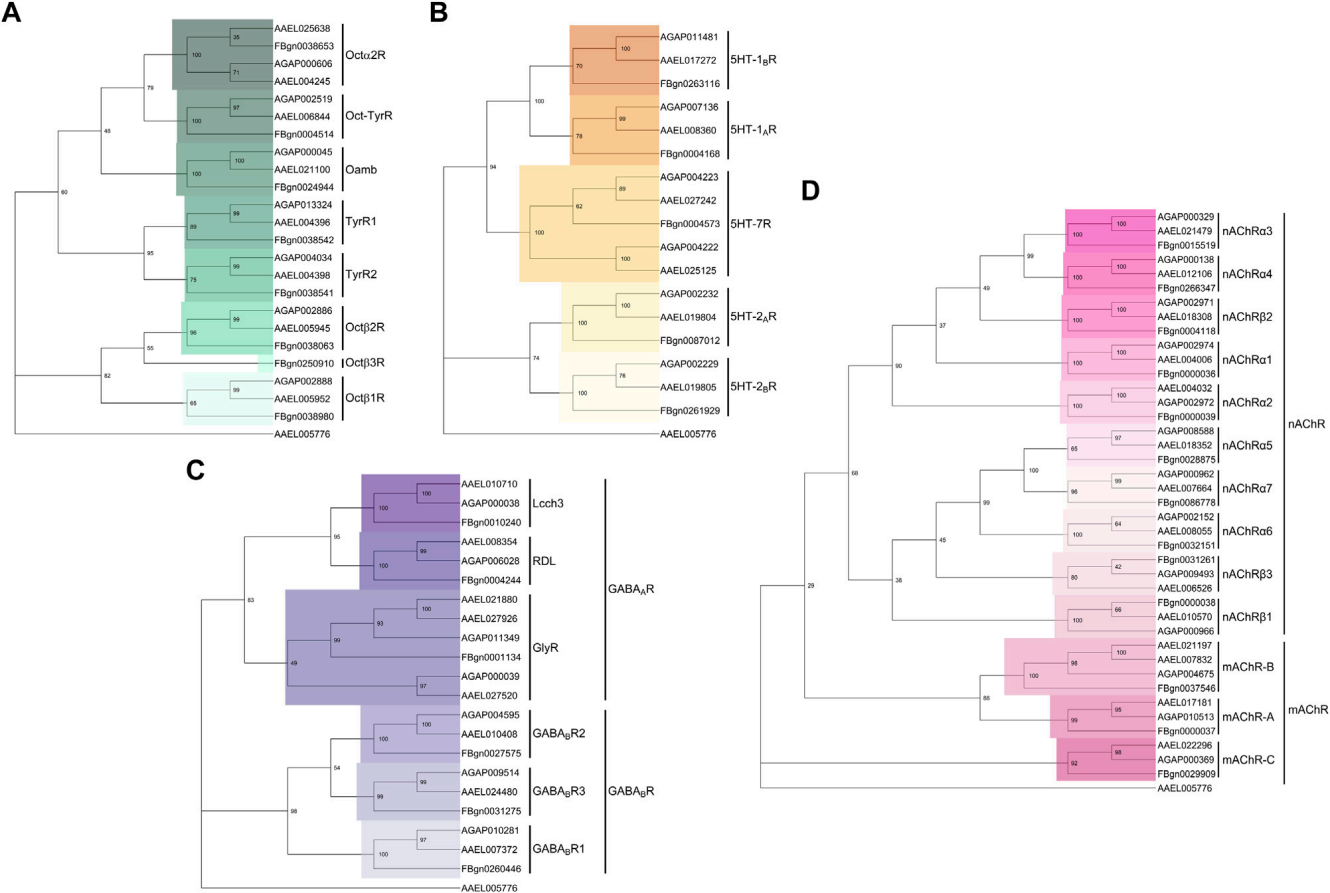
Phylogenetic trees of neurotransmitter receptors across Dipteran species. **(A–D)** Phylogenetic trees built from the protein sequence alignment for **(A)** octopamine, **(B)** serotonin, **(C)** GABA and **(D)** acetylcholine receptor subunits in *An. gambiae*, *Ae. aegypti* and *D. melanogaster*. Using MEGA (software version 11), protein sequences were first aligned using MUltiple Sequence Comparison by Log- Expectation (MUSCLE), aligned sequences were then subjected to 1000-fold bootstrapping to build the phylogenetic tree for each neurotransmitter receptor family. The *Ae. aegypti* odorant receptor obligate co-receptor *Orco* (AAEL005776) was selected as an outgroup gene.

Recent work indicates that in *An. gambiae*, octopamine appears to also play a conserved role in influencing mosquito hearing function in a sexually dimorphic and circadian time-dependent manner (Georgiades et al., 2022). Multiple homologs of *Drosophila* octopamine receptors were identified in the *An. gambiae* JO (Figure 5A). Exposing male mosquitoes to octopamine altered the flagellar mechanics of *Anopheles* males at multiple levels, including flagellar fibrillae erection (fibrillae are permanently erected in *Cx. quinquefasciatus* so this was not investigated in the previous study), a significant upward shift of the flagellar mechanical tuning frequency and an increase in flagellar stiffness values. The sensitivity of the male ear to octopamine exposure changed throughout the day, with male flagellar mechanics appearing more prone to changes (and showing a larger extent of change) upon octopamine administration during swarming, as compared to non-swarming times.

Interestingly, AGAP002886, the octopamine β2 receptor (Octβ2R) ortholog in *An. gambiae* (Figure 5A), was identified as the key octopamine receptor mediating the auditory responses to octopamine administration. *AGAP002886* ^*−/−*^ males show defects in the erection of flagellar fibrillae during swarming time and octopamine injections failed to rescue such defects. Building onto this, these mutant males also failed to respond to exogenous octopamine in terms of hearing function, showing almost unaltered mechanical tuning frequency upon octopamine exposure.

In *Drosophila*, the activation of Octβ2R by octopamine elevates intracellular cAMP levels in neurons (Lim et al., 2014). Octβ2R, which is itself an autoreceptor, reinforces octopamine signalling by switching on the cAMP and CREB-dependent autoregulatory positive feedback mechanism. It would be interesting in the future to examine if the mosquito Octβ2R AGAP002886 similarly possess autoreception properties, and if so, how the efferent octopaminergic fibres use such autoregulatory mechanism to alter the hearing function of mosquitoes.

Differential levels of octopamine and tyramine in the insect nervous system have been implicated in regulating important insect behaviours (thus far largely in *Drosophila*) such as olfaction, locomotion, feeding, and mating (Roeder, 2005; Yang et al., 2015). The antagonistic actions of octopamine and tyramine (the intermediate product of the octopamine biosynthesis pathway) have been reported to coordinate the switch in olfactory preference between gregarious and solitary locusts (Ma et al., 2015). Future work looking into the role of tyramine in addition to octopamine would also provide insights as to whether the time-dependent modulations of the hearing system of mosquitoes can be attributed to the antagonist actions of octopamine and tyramine.

### Serotonin

As another key biogenic amine, serotonin (also known as 5-hydroxytryptamine or 5-HT) regulates different aspects of animal behaviours by acting mostly *via* GPCRs (Tierney, 2018). In *D. melanogaster*, differential binding of serotonin to distinct serotonin receptors has been implicated in mediating behaviours such as mating, aggression, sleeping, feeding and circadian entrainment (Page, 1987; Yuan et al., 2005; Becnel et al., 2011; Pooryasin and Fiala, 2015; Sun et al., 2020). Five distinct serotonin receptors, split across 3 families exist in *D. melanogaster*, though further 5-HT7 family members have been found in both *Ae. aegypti* and *An. gambiae* (Fuchs et al., 2014; Ling and Raikhel, 2018; Ngai et al., 2019; Xu et al., 2022) (Figure 5B)*.*

The presence of serotonin in the ears of *Ae. aegypti* and *An. gambiae* was reported over a decade ago (Siju et al., 2008), with identification in the male *Cx. quinquesfasciatus* ear following in 2016 (Andrés et al., 2016). In *Cx. quinquefasciatus* and *Ae. aegypti* male mosquitoes, serotonin is released from S and A efferent terminals in the JO (Xu et al., 2022). The distribution pattern of serotonin signals in the female *Ae. aegypti* JO also appear to be highly similar to their male counterparts, with the only major difference being a far greater extent of co-localization between serotonin and the presynaptic marker 3C11 in the somata of females than males, suggesting different actions of mechanisms of serotonin in both sexes (Xu et al., 2022). The apparent minimal co-localization between 3C11 and serotonin signals in males yet an abundant serotonin distribution in the male somata could be attributed to local serotonin production within the male and female *Ae. aegypti* JO neurons, as demonstrated by both males and females expressing the key serotonin synthesis enzyme, *tryptophan hydroxylase* (*TPH*) in their JOs, which is also indicative of JO neurons themselves being serotonergic (Xu et al., 2022). The pattern of serotonin efferent innervation is different to octopamine, suggesting distinct and specific mechanisms of action.

Indeed, functional studies of the *Ae. aegypti* ear upon serotonin exposure support its role as an efferent neuromodulator, with the mechanical tuning frequency of the male ear increased by far greater an amount than females upon serotonin exposure (Xu et al., 2022) (Table 2). However, it is still interesting that the female tuning frequency mildly shifted given the relative non-dynamic nature of the female flagellar mechanics compared to the males (Su et al., 2018). Interestingly, inhibition of serotonin synthesis *via* alpha-methyltryptophan (AMTP) exposure leads to a reduction in the male ear mechanical tuning frequency as well as a change in male hearing behavior. As receptors from the 5-HT7 family show higher expression in the ears of *Ae. aegypti* mosquitoes in relation to the brain (Georgiades et al., 2022; Xu et al., 2022), these receptors have been proposed to be the primary molecular target of serotonin signalling in the ears and their activation or inhibition may lead to downstream changes in the hearing function.

### GABA

GABA, the major inhibitory neurotransmitter found in the nervous systems of vertebrates and invertebrates serves key roles in many physiological processes. For instance, GABA plays a conserved role in regulating the sleep-wake cycle of mammals and insects (Ono et al., 2018; Ki and Lim, 2019; Massah et al., 2022). Fluctuating levels of GABA in clock neurons are known to set the rhythmicity of activity profiles in insects (Ki and Lim, 2019; Massah et al., 2022). GABA differentially regulates neuronal excitability by binding to two structurally- and functionally-distinct receptor families, namely, GABA_A_, ligand-gated chloride ion channels and GABA_B_, a metabotropic G-protein coupled receptors (Bowery and Smart, 2006). Figure 5C shows the conservation of these receptor families in mosquitoes and *Drosophila*. Owing to the distinct receptor kinetics and mechanisms of action of these two receptor families, the effect of inhibitory GABAergic input on the target neurons may differ depending on the GABA receptor profiles that they display (Bowery and Smart, 2006).

GABAergic innervation of the nerve bundles (type I terminals) axons of JO neurons in the male *Cx. quinquefasciatus* ears has been demonstrated *via* staining of the male ears with glutamic acid decarboxylase (GAD) (Figure 4D), an enzyme responsible for the conversion of glutamate into GABA (Buddhala et al., 2009). The central origin of such GABAergic innervation is supported by the absence of GAD signals in the JO cell bodies, supporting the idea that JO neurons themselves are likely not GABAergic. In line with the anatomical evidence, functional studies have demonstrated that picrotoxin-mediated blocking of GABA_A_ receptors in *Cx. quinquefasciatus* males leads to an increase in the mechanical frequency tuning as well shifts in the DC response of afferent action potential signalling from auditory neurons (Andrés et al., 2016), hinting at the possibility of GABAergic influence on the peripheral auditory frequency tuning and first-level acoustic information processing of auditory electrical signals within JO neurons.

Potential GABA receptors in the JO that may be receiving efferent GABAergic inputs have recently been identified in the ears of *An. gambiae* (Georgiades et al., 2022). Building parallels to the GABAergic innervation of AN terminals in the JO of male *Cx. quinquefasciatus* (Andrés et al., 2016), GABA receptors are most likely located in the axonal membrane along the auditory nerve in both *Cx. quinquefasciatus* and the malaria mosquitoes. It would be interesting to study how potential combinations of different GABA receptors determine inhibitory signals of the auditory afferent information in mosquitoes. The inhibitory GABAergic system has also been implicated as serving key roles in the central processing of auditory signals (Lin and Feng, 2003; Pinaud et al., 2008; Lai et al., 2012). In *D. melanogaster*, the tuning of a subset of higher order auditory circuits to sound relies in part on GABA. GABAergic systems have also been found to play roles in song preference learning in *D. melanogaster*, with expression of the GABA_A_ receptor Resistance to dieldrin (Rdl) in pC1 neurons essential for learning to select conspecific songs from heterospecific sounds (Li et al., 2018). Rdl may thus play a similar role in auditory learning processes in mosquitoes.

Given the abundance of SVs-enriched type I presynaptic terminals that come into contact with the JO nerve bundles of male *Cx. quinquefasciatus* (Andrés et al., 2016) it would also be relevant to investigate whether, in addition to GABA, other amino-acid neurotransmitters such as glycine may also innervate this region and thus influence processing of auditory electrical signals. Investigating potential glycinergic innervations of JO nerve bundles would be an interesting approach, as previous work identified close clustering of glycinergic and GABAergic receptors at inhibitory postsynaptic sites (Groeneweg et al., 2018). In addition to the chief inhibitory neurotransmitter GABA, glutamate, the precursor of GABA and a key excitatory neurotransmitter itself, also has receptor counterparts identified in the ears of *An. gambiae* (Georgiades et al., 2022). The presence of both inhibitory GABAergic receptors and excitatory glutamatergic receptors in the JO suggests that this pair of antagonistic neurotransmitters may interact to influence JO neuron properties.

### Other neurotransmitter families

Beyond the aforementioned three major neurotransmitter types, a multitude of other families also exist, though no published studies describe their function in modulating mosquito auditory efferent systems. JO neurons across *Drosophila* species are highly cholinergic (Sivan-Loukianova and Eberl, 2005). Equivalent studies in mosquitoes are lacking, but based on an analysis of the recent *An. gambiae* JO RNAseq dataset many cholinergic receptor subunits are expressed in the mosquito JO (Georgiades et al., 2022), suggesting that ACh might be involved in both the afferent and efferent auditory signalling pathways in mosquitoes. Targeting individual subunits of the cholinergic receptors might shed light on its auditory role, though this may be challenging given the plethora of existing receptors and potential redundant roles (Figure 5D).

The importance of song-dependent learning in *D. melanogaster*, and the extensive innervation of multiple brain regions (including AL) in mosquitoes by tyrosine hydroxylase (Wolff et al., 2023), suggest that dopamine may play a central role in terms of auditory information processing. Given the crucial role of the dopaminergic system in mediating learning and memory formation, the involvement of dopamine in mosquito auditory-related learning processes may be of interest for future investigations (Berry et al., 2012).

## Comparison between invertebrate and vertebrate efferent systems

Whilst auditory efferent systems have not been reported in insects outside of mosquito species (though see previous discussion on *D. melanogaster*), all vertebrates show descending efferent control of their hearing. Efferent innervation has been described across reptile, fish, avian and mammalian species (Manley, 2000). In most vertebrates, the main auditory efferent activity is cholinergic and controls the sensitivity of the auditory system (Guinan, 2018; Lopez-Poveda, 2018).

In mammals, central control of peripheral hearing function is mediated *via* olivocochlear efferent neurons that originate in the superior olivary complexes of the brain and project to the cochlea (Ryugo et al., 2010). They split into two groups depending on whether they start from medial or lateral sides: medial olivocochlear (MOC) neurons that innervate outer hair cells (OHC) and lateral olivocochlear (LOC) neurons that synapse on afferent fibers innervating inner hair cells. Both groups of efferent neurons utilise ACh as the major neurotransmitter (Ryugo et al., 2010) although other neurotransmitters have been described mostly in LOC. Despite obvious differences across invertebrate and vertebrate ears, there are common structural and functional principles shared across their auditory efferent systems (Albert and Kozlov, 2016).

A common and integral feature of invertebrate and vertebrate hearing is an active and intensity-dependent mechanical amplification of sound, which has been shown to be controlled by efferent activity. In mammals, MOC efferent innervation on OHC turns down the gain of the cochlear amplifier and affects Prestin-mediated OHC electromotility (He et al., 2003; Guinan, 2018). These mechanisms have been linked to aiding in the discrimination of sound in noisy environments and protecting the ear from acoustic trauma. In mosquitoes, efferent control also affects the mechanical amplification of sounds and reduce the extent of flagellar mechanical responses induced by sound stimulation (Su et al., 2018; Georgiades et al., 2022). It is worth noting that a modulation of signal-to-noise ratio by efferent control could be essential in the sensory context of the swarm, where hundreds of mosquitoes fly together. The swarm is a highly noisy and dynamic environment, and its sensory ecology can greatly vary depending on a number of factors such as changes in mosquito numbers, temperature or wind. The detection of the faint female flight tones by males in this challenging context might have acted as a selection pressure to drive the emergence of an efferent network to control the mosquito auditory system, thus enhancing the performance and plasticity of male hearing. It would be worth exploring if other insects that mate in swarms and rely on the acoustic detection of mating partner, such as midges (Fedorova and Zhantiev, 2009) also present efferent innervation of their ears, perhaps establishing an association between the acoustic ecology of the swarms and the presence of auditory efferent systems.

There seems to be a convergence in the effects of the efferent innervation in controlling the ear’s spontaneous activity across vertebrates and mosquitoes. SSOs, which much like SOAEs in vertebrates are the most impressive signature of mechanical feedback amplification in mosquitoes, disappear when efferent signalling is ablated, suggesting that in addition to having a transducer-based mechanism, the auditory efferent system of males also acts concertedly to control the male ear mechanical amplification (Su et al., 2018). Recent work in male *An. gambiae* shows that exogenous octopamine administration mimicking efferent octopamine release into the JO causes an increase in SSO frequency coupled to a decrease in amplitude that ultimately leads to SSO cessation, likely mediated through an increase in the flagellar stiffness (Georgiades et al., 2022). Likewise, the effect of MOC on SOAEs is a reduction of their amplitude and a shift to higher frequencies (Zhao and Dhar, 2011; Zhao et al., 2015).

From a neurochemical perspective, MOC fibers release ACh onto OHC. In this system, ACh plays an inhibitory role mediated by the α9α10 nicotinic cholinergic receptor (α9α10 nAChR), which is highly permeable to Ca^2+^, that in-turn activates K^+^ channels to hyperpolarize OHC (Glowatzki and Fuchs, 2000; Oliver et al., 2000). In *An. gambiae* mosquitoes, transcriptomic analysis found evidence of multiple α nAChR subunits being expressed in the JO (Georgiades et al., 2022), although its function remains elusive. It is worth noting that GABA co-localizes with ACh in MOC terminals, suggesting a putative modulation of ACh release by GABA (Maison et al., 2003, 2006; Katz and Elgoyhen, 2014). In mosquitoes, there is large GABAergic innervation of the auditory nerve (Figure 4D) that inhibits sound-induced electrical responses of the auditory nerve (Andrés et al., 2016). A better understanding of GABA functions in both systems could help to establish parallel methods of inhibitory auditory control. Although LOC efferent function is less understood, different reports show that apart from ACh, LOC terminals also release other neurotransmitters such as GABA (Plinkert et al., 1993), serotonin (Gil-Loyzaga et al., 1997), dopamine (Wu et al., 2020), calcitonin gene-related peptide (CGRP) (Le Prell et al., 2021) and other opiod peptides (Safieddine et al., 1997; Lioudyno et al., 2002). Interestingly, an ortholog of CGRP receptor has been found to be expressed in the *An. gambiae* JO (Georgiades et al., 2022).

Biogenic amines have been shown to play an essential role in the efferent modulation of mosquito hearing (Georgiades et al., 2022; Xu et al., 2022). Octopamine’s effect on the auditory tuning of the male mosquito is circadian-time dependent and has been linked to increase the female audibility in the swarm (Georgiades et al., 2022). In mammals, auditory function is affected by circadian rhythms (Meltser et al., 2014; Basinou et al., 2017), although the efferent system has not been yet implicated in this regulation. However, the mechanisms identified to be under circadian-clock control in the cochlea include the sensitivity to noise trauma (Meltser et al., 2014) and the frequency of SOAEs (Haggerty et al., 1993; Cacace et al., 1996), both of which are linked to the MOC efferent activity. It would be interesting to explore whether the auditory efferent system confers circadian rhythmicity to peripheral cochlear function.

## Conclusion

Understanding the physiology and function of the mosquito auditory efferent system raises many tantalising questions. The presence of efferent innervation in evolutionarily diverse mosquito species suggests that it is an intrinsic component of the mosquito auditory system that may be linked to boosting relevant sound detection in the sensory context of male-dominated swarms. The diversity in neurotransmitters, modes of action, sites of release and target cell-binding suggest complex modulatory patterns comparable to auditory efferent systems in vertebrates. Understanding the effects of efferent activity on the physiology of the mosquito ear can illustrate basic biophysical principles inherent to the auditory function in a system far more accessible than their vertebrate counterparts. More research is required to enable the molecular and genetic dissection of these processes that can potentially inform research in more complex mammalian and human systems. Moreover, since accurate auditory efferent control is linked to mosquito mating fitness, the receptors might offer new and exciting opportunities for the control of disease-transmitting mosquito populations.

## Abbreviations

5-HT, 5-hydroxytryptamine; ACh, acetylcholine; *Ae. aegypti*, *Aedes aegypti*; *An. gambiae*, *Anopheles gambiae*; AL, antennal lobe; AMMC, antennal mechanosensory and motor center; AMTP, alphamethyltrytophan; AN, auditory nerve; BP, basal plate; CB, ciliary base; CGRP, calcitonin gene-related peptide; *Cx. quinquefasciatus*, *Culex quinquefasciatus*; DCVs, dense-core vesicles; *D. melanogaster*, *Drosophila melanogaster*; DMSO, dimethyl sulfoxide; GABA, gamma-aminobutyric acid; GAD, glutamic acid decarboxylase; GPCRs, G-protein-coupled receptors; JO, Johnston’s organ; JOC, Johnston’s organ centre; DCVs, dense-core vesicles; LDV, laser Doppler vibrometry; LOC, lateral olivocochlear; MOC, medial olivocochlear; OHCs, outer hair cells; Rdl, Resistance to dieldrin; SVs, synaptic vesicles; SOAEs, spontaneous otoacoustic emissions; SSOs, self-sustained oscillations; TEM, transmission electron microscopy; TPH, tryptophan hydroxylase.

## References

[B1] Albert J. T. Kozlov A. S. (2016). Comparative aspects of hearing in vertebrates and insects with antennal ears. Curr. Biol. 26, R1050–r1061. 10.1016/j.cub.2016.09.017 27780047

[B2] Andrés M. Seifert M. Spalthoff C. Warren B. Weiss L. Giraldo D. (2016). Auditory efferent system modulates mosquito hearing. Curr. Biol. 26, 2028–2036. 10.1016/j.cub.2016.05.077 27476597

[B3] Andrés M. Su M. P. Albert J. Cator L. J. (2020). Buzzkill: Targeting the mosquito auditory system. Curr. Opin. Insect Sci. 40, 11–17. 10.1016/j.cois.2020.04.003 32505906

[B4] Ashmore J. Avan P. Brownell W. E. Dallos P. Dierkes K. Fettiplace R. (2010). The remarkable cochlear amplifier. Hear Res. 266, 1–17. 10.1016/j.heares.2010.05.001 20541061 PMC6366996

[B5] Bartlett-Healy K. Crans W. Gaugler R. (2008). Phonotaxis to Amphibian vocalizations in *Culex territans* (Diptera: Culicidae). Ann. Entomol. Soc. Am. 101, 95–103. 10.1603/0013-8746(2008)101[95:PTAVIC]2.0.CO;2

[B6] Basinou V. Park J.-S. Cederroth C. R. Canlon B. (2017). Circadian regulation of auditory function. Hear Res. 347, 47–55. 10.1016/j.heares.2016.08.018 27665709 PMC5364078

[B7] Becnel J. Johnson O. Luo J. Nässel D. R. Nichols C. D. (2011). The serotonin 5-HT_7_Dro receptor is expressed in the brain of *Drosophila*, and is essential for normal courtship and mating. PLoS One 6, e20800. 10.1371/journal.pone.0020800 21674056 PMC3107233

[B8] Belton P. (1994). Attraction of male mosquitoes to sound. J. Am. Mosq. Control 10, 297–301. 8965082

[B9] Berry J. A. Cervantes-Sandoval I. Nicholas E. P. Davis R. L. (2012). Dopamine is required for learning and forgetting in *Drosophila* . Neuron 74, 530–542. 10.1016/j.neuron.2012.04.007 22578504 PMC4083655

[B10] Boo K. S. (1980). Antennal sensory receptors of the male mosquito, *Anopheles stephensi* . Z. für Parasitenkd. 61, 249–264. 10.1007/BF00925516 7368776

[B11] Boo K. S. Richards A. G. (1975a). Fine structure of scolopidia in Johnston’s organ of female *Aedes aegypti* compared with that of the male. J. Insect Physiology 21, 1129–1139. 10.1016/0022-1910(75)90126-2 1141704

[B12] Boo K. S. Richards A. G. (1975b). Fine structure of the scolopidia in the Johnston’s organ of male *Aedes aegypti* (L.)(Diptera: Culicidae). Int. J. Insect Morphol. Embryology 4, 549–566. 10.1016/0020-7322(75)90031-8 1141704

[B13] Bowery N. G. Smart T. G. (2006). GABA and glycine as neurotransmitters: A brief history. Br. J. Pharmacol. 147, S109–S119. 10.1038/sj.bjp.0706443 16402094 PMC1760744

[B14] Buddhala C. Hsu C.-C. Wu J.-Y. (2009). A novel mechanism for GABA synthesis and packaging into synaptic vesicles. Neurochem. Int. 55, 9–12. 10.1016/j.neuint.2009.01.020 19428801

[B15] Cacace A. T. McClelland W. A. Weiner J. McFarland D. J. (1996). Individual differences and the reliability of 2F1-F2 distortion-product otoacoustic emissions: Effects of time-of-day, stimulus variables, and gender. J. Speech Hear Res. 39, 1138–1148. 10.1044/jshr.3906.1138 8959599

[B16] Clements A. N. (1999). The biology of mosquitoes. Sensory reception and behaviour. Wallingford: CABI Publishing Google Scholar.

[B17] Coutinho-Abreu I. V. Akbari O. S. (2022). Technological advances in mosquito olfaction neurogenetics. *Trends Genet.* 0 39, 154–166. 10.1016/j.tig.2022.10.007 PMC10564117 36414481

[B18] da Silva A. F. Machado L. C. de Paula M. B. da Silva Pessoa Vieira C. J. de Morais Bronzoni R. V. de Melo Santos M. A. V. (2020). Culicidae evolutionary history focusing on the Culicinae subfamily based on mitochondrial phylogenomics. Sci. Rep. 10, 18823. 10.1038/s41598-020-74883-3 33139764 PMC7606482

[B19] Dallos P. (1992). The active cochlea. J. Neurosci. 12, 4575–4585. 10.1523/JNEUROSCI.12-12-04575.1992 1464757 PMC6575778

[B20] Diabate A. Tripet F. (2015). Targeting male mosquito mating behaviour for malaria control. Parasites Vectors 8, 347. 10.1186/s13071-015-0961-8 26113015 PMC4485859

[B21] Eberl D. F. Boekhoff-Falk G. (2007). Development of Johnston’s organ in *Drosophila* . Int. J. Dev. Biol. 51, 679–687. 10.1387/ijdb.072364de 17891726 PMC3417114

[B22] Edwards R. H. (1998). Neurotransmitter release: Variations on a theme. Curr. Biol. 8, R883–R885. 10.1016/S0960-9822(07)00551-9 9843673

[B23] Farooqui T. (2007). Octopamine-mediated neuromodulation of insect senses. Neurochem. Res. 32, 1511–1529. 10.1007/s11064-007-9344-7 17484052

[B24] Fedorova M. V. Zhantiev R. D. (2009). Structure and function of the Johnston’s organ in *Fleuria lacustris* Kieff. males (Diptera, Chironomidae). Entomol. Rev. 89, 896–902. 10.1134/s001387380908003x

[B25] Fettiplace R. Hackney C. M. (2006). The sensory and motor roles of auditory hair cells. Nat. Rev. Neurosci. 7, 19–29. 10.1038/nrn1828 16371947

[B26] Field L. H. Matheson T. (1998). “Chordotonal organs of insects,” in Advances in insect physiology. Editor Evans P. D. (Academic Press), 1–228. 10.1016/S0065-2806(08)60013-2

[B27] Flecke C. Stengl M. (2009). Octopamine and tyramine modulate pheromone-sensitive olfactory sensilla of the hawkmoth *Manduca sexta* in a time-dependent manner. J. Comp. Physiology A 195, 529–545. 10.1007/s00359-009-0429-4 19301013

[B28] Fuchs S. Rende E. Crisanti A. Nolan T. (2014). Disruption of aminergic signalling reveals novel compounds with distinct inhibitory effects on mosquito reproduction, locomotor function and survival. Sci. Rep. 4, 5526. 10.1038/srep05526 24984706 PMC4078307

[B29] Fuxe K. Dahlström A. B. Jonsson G. Marcellino D. Guescini M. Dam M. (2010). The discovery of central monoamine neurons gave volume transmission to the wired brain. Prog. Neurobiol. 90, 82–100. 10.1016/j.pneurobio.2009.10.012 19853007

[B30] Georgiades M. Alampounti C. A. Somers J. Su M. Ellis D. Bagi J. (2022). A novel beta-adrenergic like octopamine receptor modulates the audition of malaria mosquitoes and serves as insecticide target. bioRxiv 08, 502538. 10.1101/2022.08.02.502538 PMC10356864 37468470

[B31] Gil-Loyzaga P. Bartolomé M. V. Vicente-Torres M. A. (1997). Serotonergic innervation of the organ of Corti of the cat cochlea. Neuroreport 8, 3519–3522. 10.1097/00001756-199711100-00020 9427318

[B32] Glowatzki E. Fuchs P. A. (2000). Cholinergic synaptic inhibition of inner hair cells in the neonatal mammalian cochlea. Science 288, 2366–2368. 10.1126/science.288.5475.2366 10875922

[B33] Göpfert M. C. Albert J. T. (2006). “Mechanical energy contributed by motile neurons in the *Drosophila* ear,” in Auditory mechanisms: Processes and models (World Scientific), 489–495. 10.1142/9789812773456_0076

[B34] Göpfert M. C. Briegel H. Robert D. (1999). Mosquito hearing: Sound-induced antennal vibrations in male and female *Aedes aegypti* . J. Exp. Biol. 202, 2727–2738. 10.1242/jeb.202.20.2727 10504309

[B35] Göpfert M. C. Robert D. (2001). Active auditory mechanics in mosquitoes. Proc. Biol. Sci. 268, 333–339. 10.1098/rspb.2000.1376 11270428 PMC1088611

[B36] Göpfert M. C. Robert D. (2003). Motion generation by *Drosophila* mechanosensory neurons. Proc. Natl. Acad. Sci. 100, 5514–5519. 10.1073/pnas.0737564100 12642657 PMC154376

[B37] Göpfert M. C. Robert D. (2000). Nanometre–range acoustic sensitivity in male and female mosquitoes. Proc. R. Soc. Lond. B Biol. Sci. 267, 453–457. 10.1098/rspb.2000.1021 PMC1690551 10737401

[B38] Gregor K. M. Becker S. C. Hellhammer F. Baumgärtner W. Puff C. (2022). Immunohistochemical characterization of the nervous system of *Culex pipiens* (Diptera, Culicidae). Biol. (Basel) 11, 57. 10.3390/biology11010057 PMC8772823 35053056

[B39] Groeneweg F. L. Trattnig C. Kuhse J. Nawrotzki R. A. Kirsch J. (2018). Gephyrin: A key regulatory protein of inhibitory synapses and beyond. Histochem Cell Biol. 150, 489–508. 10.1007/s00418-018-1725-2 30264265

[B40] Guidolin D. Tortorella C. Marcoli M. Maura G. Agnati L. F. (2022). Intercellular communication in the central nervous system as deduced by chemical neuroanatomy and quantitative analysis of images: Impact on neuropharmacology. Int. J. Mol. Sci. 23, 5805. 10.3390/ijms23105805 35628615 PMC9145073

[B41] Guinan J. J. (2018). Olivocochlear efferents: Their action, effects, measurement and uses, and the impact of the new conception of cochlear mechanical responses. Hear. Res. 362, 38–47. 10.1016/j.heares.2017.12.012 29291948 PMC5911200

[B42] Haggerty H. S. Lusted H. S. Morton S. C. (1993). Statistical quantification of 24-hour and monthly variabilities of spontaneous otoacoustic emission frequency in humans. Hear Res. 70, 31–49. 10.1016/0378-5955(93)90050-b 8276731

[B43] Hampel S. Eichler K. Yamada D. Bock D. D. Kamikouchi A. Seeds A. M. (2020). Distinct subpopulations of mechanosensory chordotonal organ neurons elicit grooming of the fruit fly antennae. eLife 9, e59976. 10.7554/eLife.59976 33103999 PMC7652415

[B44] He D. Z. Z. Jia S. Dallos P. (2003). Prestin and the dynamic stiffness of cochlear outer hair cells. J. Neurosci. 23, 9089–9096. 10.1523/JNEUROSCI.23-27-09089.2003 14534242 PMC6740818

[B45] Herre M. Goldman O. V. Lu T.-C. Caballero-Vidal G. Qi Y. Gilbert Z. N. (2022). Non-canonical odor coding in the mosquito. Cell 185, 3104–3123.e28. 10.1016/j.cell.2022.07.024 35985288 PMC9480278

[B46] Hudspeth A. (1997). Mechanical amplification of stimuli by hair cells. Curr. Opin. Neurobiol. 7, 480–486. 10.1016/S0959-4388(97)80026-8 9287199

[B47] Ignell R. Dekker T. Ghaninia M. Hansson B. S. (2005). Neuronal architecture of the mosquito deutocerebrum. J. Comp. Neurol. 493, 207–240. 10.1002/cne.20800 16255032

[B48] Kamikouchi A. Albert J. T. Göpfert M. C. (2010). Mechanical feedback amplification in *Drosophila* hearing is independent of synaptic transmission. Eur. J. Neurosci. 31, 697–703. 10.1111/j.1460-9568.2010.07099.x 20384813

[B49] Kamikouchi A. Inagaki H. K. Effertz T. Hendrich O. Fiala A. Gopfert M. C. (2009). The neural basis of *Drosophila* gravity-sensing and hearing. Nature 458, 165–171. 10.1038/nature07810 19279630

[B50] Kamikouchi A. Shimada T. Ito K. (2006). Comprehensive classification of the auditory sensory projections in the brain of the fruit fly *Drosophila melanogaster* . J. Comp. Neurol. 499, 317–356. 10.1002/cne.21075 16998934

[B51] Karak S. Jacobs J. S. Kittelmann M. Spalthoff C. Katana R. Sivan-Loukianova E. (2015). Diverse roles of axonemal dyneins in *Drosophila* auditory neuron function and mechanical amplification in hearing. Sci. Rep. 5, 17085. 10.1038/srep17085 26608786 PMC4660584

[B52] Katz E. Elgoyhen A. B. (2014). Short-term plasticity and modulation of synaptic transmission at mammalian inhibitory cholinergic olivocochlear synapses. Front. Syst. Neurosci. 8, 224. Available at: https://www.frontiersin.org/articles/10.3389/fnsys.2014.00224 . 10.3389/fnsys.2014.00224 25520631 PMC4251319

[B53] Keder A. Tardieu C. Malong L. Filia A. Kashkenbayeva A. Newton F. (2020). Homeostatic maintenance and age-related functional decline in the *Drosophila* ear. Sci. Rep. 10, 7431. 10.1038/s41598-020-64498-z 32366993 PMC7198581

[B54] Ki Y. Lim C. (2019). Sleep-promoting effects of threonine link amino acid metabolism in *Drosophila* neuron to GABAergic control of sleep drive. eLife 8, e40593. 10.7554/eLife.40593 31313987 PMC6636906

[B55] Kim H. Horigome M. Ishikawa Y. Li F. Lauritzen J. S. Card G. (2020). Wiring patterns from auditory sensory neurons to the escape and song-relay pathways in fruit flies. J. Comp. Neurol. 528, 2068–2098. 10.1002/cne.24877 32012264 PMC7676477

[B56] Konopka J. K. Task D. Poinapen D. Potter C. J. (2022). Neurogenetic identification of mosquito sensory neurons. bioRxiv 11, 517370. 10.1101/2022.11.22.517370 PMC10172775 37182106

[B57] Lai J. S.-Y. Lo S.-J. Dickson B. J. Chiang A.-S. (2012). Auditory circuit in the *Drosophila* brain. Proc. Natl. Acad. Sci. 109, 2607–2612. 10.1073/pnas.1117307109 22308412 PMC3289363

[B58] Lapshin D. N. Vorontsov D. D. (2019). Directional and frequency characteristics of auditory neurons in *Culex* male mosquitoes. J. Exp. Biol. 222, jeb208785. 10.1242/jeb.208785 31586018

[B59] Lapshin D. N. Vorontsov D. D. (2017). Frequency organization of the Johnston’s organ in male mosquitoes (Diptera, Culicidae). J. Exp. Biol. 220, 3927–3938. 10.1242/jeb.152017 28851820

[B60] Lapshin D. N. Vorontsov D. D. (2013). Frequency tuning of individual auditory receptors in female mosquitoes (Diptera, Culicidae). J. Insect Physiology 59, 828–839. 10.1016/j.jinsphys.2013.05.010 23742968

[B61] Le Prell C. G. Hughes L. F. Dolan D. F. Bledsoe S. C. (2021). Effects of calcitonin-gene-related-peptide on auditory nerve activity. Front. Cell Dev. Biol. 9, 752963. 10.3389/fcell.2021.752963 34869340 PMC8633412

[B62] Li J. Merchant A. Zhou S. Wang T. Zhou X. Zhou C. (2022). Neuroanatomical basis of sexual dimorphism in the mosquito brain. iScience 25, 105255. 10.1016/j.isci.2022.105255 36277452 PMC9583127

[B63] Li X. Ishimoto H. Kamikouchi A. (2018). Auditory experience controls the maturation of song discrimination and sexual response in *Drosophila* . eLife 7, e34348. 10.7554/eLife.34348 29555017 PMC5860867

[B64] Lim J. Sabandal P. R. Fernandez A. Sabandal J. M. Lee H.-G. Evans P. (2014). The octopamine receptor Octβ2R regulates ovulation in *Drosophila melanogaster* . PLOS ONE 9, e104441. 10.1371/journal.pone.0104441 25099506 PMC4123956

[B65] Lin W.-Y. Feng A. S. (2003). GABA is involved in spatial unmasking in the frog auditory midbrain. J. Neurosci. 23, 8143–8151. 10.1523/JNEUROSCI.23-22-08143.2003 12954877 PMC6740497

[B66] Ling L. Raikhel A. S. (2018). Serotonin signaling regulates insulin-like peptides for growth, reproduction, and metabolism in the disease vector *Aedes aegypti* . Proc. Natl. Acad. Sci. 115, E9822–E9831. 10.1073/pnas.1808243115 30275337 PMC6196551

[B67] Linn C. E. Campbell M. G. Poole K. R. Wu W.-Q. Roelofs W. L. (1996). Effects of photoperiod on the circadian timing of pheromone response in male *Trichoplusia ni*: Relationship to the modulatory action of octopamine. J. Insect Physiology 42, 881–891. 10.1016/0022-1910(96)00034-0

[B68] Lioudyno M. I. Verbitsky M. Glowatzki E. Holt J. C. Boulter J. Zadina J. E. (2002). The alpha9/alpha10-containing nicotinic ACh receptor is directly modulated by opioid peptides, endomorphin-1, and dynorphin B, proposed efferent cotransmitters in the inner ear. Mol. Cell Neurosci. 20, 695–711. 10.1006/mcne.2002.1150 12213449

[B69] Liu C. Goel P. Kaeser P. S. (2021). Spatial and temporal scales of dopamine transmission. Nat. Rev. Neurosci. 22, 345–358. 10.1038/s41583-021-00455-7 33837376 PMC8220193

[B70] Lopez-Poveda E. A. (2018). Olivocochlear efferents in animals and humans: From anatomy to clinical relevance. Front. Neurol. 9, 197. 10.3389/fneur.2018.00197 29632514 PMC5879449

[B71] Ma Z. Guo X. Lei H. Li T. Hao S. Kang L. (2015). Octopamine and tyramine respectively regulate attractive and repulsive behavior in locust phase changes. Sci. Rep. 5, 8036. 10.1038/srep08036 25623394 PMC5389030

[B72] Maison S. F. Adams J. C. Liberman M. C. (2003). Olivocochlear innervation in the mouse: Immunocytochemical maps, crossed versus uncrossed contributions, and transmitter colocalization. J. Comp. Neurology 455, 406–416. 10.1002/cne.10490 PMC1805785 12483691

[B73] Maison S. F. Rosahl T. W. Homanics G. E. Liberman M. C. (2006). Functional role of GABAergic innervation of the cochlea: Phenotypic analysis of mice lacking GABA(A) receptor subunits α1, α2, α5, α6, β2, β3, or δ. J. Neurosci. 26, 10315–10326. 10.1523/JNEUROSCI.2395-06.2006 17021187 PMC1806703

[B74] Manley G. A. (2000). Cochlear mechanisms from a phylogenetic viewpoint. Proc. Natl. Acad. Sci. U. S. A. 97, 11736–11743. 10.1073/pnas.97.22.11736 11050203 PMC34343

[B75] Massah A. Neupert S. Brodesser S. Homberg U. Stengl M. (2022). Distribution and daily oscillation of GABA in the circadian system of the cockroach *Rhyparobia maderae* . J. Comp. Neurology 530, 770–791. 10.1002/cne.25244 34586642

[B76] Matsuo E. Yamada D. Ishikawa Y. Asai T. Ishimoto H. Kamikouchi A. (2014). Identification of novel vibration- and deflection-sensitive neuronal subgroups in Johnston’s organ of the fruit fly. Front. Physiol. 5, 179. 10.3389/fphys.2014.00179 24847281 PMC4023023

[B77] Meltser I. Cederroth C. R. Basinou V. Savelyev S. Lundkvist G. S. Canlon B. (2014). TrkB-mediated protection against circadian sensitivity to noise trauma in the murine cochlea. Curr. Biol. 24, 658–663. 10.1016/j.cub.2014.01.047 24583017 PMC3962718

[B78] Mhatre N. (2015). Active amplification in insect ears: Mechanics, models and molecules. J. Comp. Physiol. A 201, 19–37. 10.1007/s00359-014-0969-0 25502323

[B79] Ngai M. Shoue D. A. Loh Z. McDowell M. A. (2019). The pharmacological and functional characterization of the serotonergic system in *Anopheles gambiae* and *Aedes aegypti*: Influences on flight and blood-feeding behavior. Sci. Rep. 9, 4421. 10.1038/s41598-019-38806-1 30872615 PMC6418270

[B80] Oliver D. He D. Z. Kloecker N. Ludwig J. Schulte U. Waldegger S. (2001). Intracellular anions as the voltage sensor of prestin, the outer hair cell motor protein. Science 292, 2340–2343. 10.1126/science.1060939 11423665

[B81] Oliver D. Klöcker N. Schuck J. Baukrowitz T. Ruppersberg J. P. Fakler B. (2000). Gating of Ca2+-activated K+ channels controls fast inhibitory synaptic transmission at auditory outer hair cells. Neuron 26, 595–601. 10.1016/s0896-6273(00)81197-6 10896156

[B82] Ono D. Honma K. Yanagawa Y. Yamanaka A. Honma S. (2018). Role of GABA in the regulation of the central circadian clock of the suprachiasmatic nucleus. J. Physiol. Sci. 68, 333–343. 10.1007/s12576-018-0604-x 29560549 PMC10717195

[B83] Page T. L. (1987). Serotonin phase-shifts the circadian rhythm of locomotor activity in the cockroach. J. Biol. Rhythms 2, 23–34. 10.1177/074873048700200103 2979649

[B84] Pennetier C. Warren B. Dabiré K. R. Russell I. J. Gibson G. (2010). Singing on the wing” as a mechanism for species recognition in the malarial mosquito *Anopheles gambiae* . Curr. Biol. 20, 131–136. 10.1016/j.cub.2009.11.040 20045329

[B85] Pinaud R. Terleph T. A. Tremere L. A. Phan M. L. Dagostin A. A. Leão R. M. (2008). Inhibitory network interactions shape the auditory processing of natural communication signals in the songbird auditory forebrain. J. Neurophysiology 100, 441–455. 10.1152/jn.01239.2007 18480371 PMC2493480

[B86] Plinkert P. K. Gitter A. H. Möhler H. Zenner H. P. (1993). Structure, pharmacology and function of GABA-A receptors in cochlear outer hair cells. Eur. Arch. Otorhinolaryngol. 250, 351–357. 10.1007/BF00188385 8260146

[B87] Pooryasin A. Fiala A. (2015). Identified serotonin-releasing neurons induce behavioral quiescence and suppress mating in *Drosophila* . J. Neurosci. 35, 12792–12812. 10.1523/JNEUROSCI.1638-15.2015 26377467 PMC6795202

[B88] Roeder T. (2005). Tyramine and octopamine: Ruling behavior and metabolism. Annu. Rev. Entomol. 50, 447–477. 10.1146/annurev.ento.50.071803.130404 15355245

[B89] Roth L. M. (1948). A study of mosquito behavior. An experimental laboratory study of the sexual behavior of *Aedes aegypti* (Linnaeus). Am. Midl. Nat. 40, 265–352. 10.2307/2421604

[B90] Ryugo D. K. Fay R. R. Popper A. N. (2010). Auditory and vestibular efferents. New York: Springer Science & Business Media.

[B91] Safieddine S. Prior A. M. Eybalin M. (1997). Choline acetyltransferase, glutamate decarboxylase, tyrosine hydroxylase, calcitonin gene-related peptide and opioid peptides coexist in lateral efferent neurons of rat and Guinea-pig. Eur. J. Neurosci. 9, 356–367. 10.1111/j.1460-9568.1997.tb01405.x 9058055

[B92] Sawadogo S. P. Niang A. Bilgo E. Millogo A. Maïga H. Dabire R. K. (2017). Targeting male mosquito swarms to control malaria vector density. PLOS ONE 12, e0173273. 10.1371/journal.pone.0173273 28278212 PMC5344402

[B93] Schendzielorz T. Schirmer K. Stolte P. Stengl M. (2015). Octopamine regulates antennal sensory neurons via daytime-dependent changes in cAMP and IP3 levels in the hawkmoth *Manduca sexta* . PLOS ONE 10, e0121230. 10.1371/journal.pone.0121230 25785721 PMC4364694

[B94] Shankar S. McMeniman C. J. (2020). An updated antennal lobe atlas for the yellow fever mosquito *Aedes aegypti* . PLOS Neglected Trop. Dis. 14, e0008729. 10.1371/journal.pntd.0008729 PMC7575095 33079925

[B95] Siju K. P. Hansson B. S. Ignell R. (2008). Immunocytochemical localization of serotonin in the central and peripheral chemosensory system of mosquitoes. Arthropod Struct. Dev. 37, 248–259. 10.1016/j.asd.2007.12.001 18424232

[B96] Sivan-Loukianova E. Eberl D. F. (2005). Synaptic ultrastructure of *Drosophila* Johnston’s organ axon terminals as revealed by an enhancer trap. J. Comp. Neurology 491, 46–55. 10.1002/cne.20687 PMC1802124 16127697

[B97] Somers J. Georgiades M. Su M. P. Bagi J. Andrés M. Alampounti A. (2022). Hitting the right note at the right time: Circadian control of audibility in *Anopheles* mosquito mating swarms is mediated by flight tones. Sci. Adv. 8, eabl4844. 10.1126/sciadv.abl4844 35020428 PMC8754303

[B98] Stocker B. Bochow C. Damrau C. Mathejczyk T. Wolfenberg H. Colomb J. (2018). Structural and molecular properties of insect type II motor axon terminals. Front. Syst. Neurosci. 12, 5. 10.3389/fnsys.2018.00005 29615874 PMC5867341

[B99] Su M. P. Andrés M. Boyd-Gibbins N. Somers J. Albert J. T. (2018). Sex and species specific hearing mechanisms in mosquito flagellar ears. Nat. Commun. 9, 3911. 10.1038/s41467-018-06388-7 30254270 PMC6156513

[B100] Sun Y. Qiu R. Li X. Cheng Y. Gao S. Kong F. (2020). Social attraction in *Drosophila* is regulated by the mushroom body and serotonergic system. Nat. Commun. 11, 5350. 10.1038/s41467-020-19102-3 33093442 PMC7582864

[B101] Tierney A. J. (2018). Invertebrate serotonin receptors: A molecular perspective on classification and pharmacology. J. Exp. Biol. 221, jeb184838. 10.1242/jeb.184838 30287590

[B102] Warren B. Gibson G. Russell I. J. (2009). Sex Recognition through midflight mating duets in *Culex* mosquitoes is mediated by acoustic distortion. Curr. Biol. 19, 485–491. 10.1016/j.cub.2009.01.059 19269180

[B103] Warren B. Nowotny M. (2021). Bridging the gap between mammal and insect ears – a comparative and evolutionary view of sound-reception. Front. Ecol. Evol. 9. Available at: https://www.frontiersin.org/articles/10.3389/fevo.2021.667218 . 10.3389/fevo.2021.667218

[B104] Wolff G. H. Lahondère C. Vinauger C. Rylance E. Riffell J. A. (2023). Neuromodulation and differential learning across mosquito species. Proc. R. Soc. B Biol. Sci. 290, 20222118. 10.1098/rspb.2022.2118 PMC9832544 36629098

[B105] Wu J. S. Yi E. Manca M. Javaid H. Lauer A. M. Glowatzki E. (2020). Sound exposure dynamically induces dopamine synthesis in cholinergic LOC efferents for feedback to auditory nerve fibers. eLife 9, e52419. 10.7554/eLife.52419 31975688 PMC7043886

[B106] Xu Y. Y. J. Loh Y. M. Lee T.-T. Ohashi T. S. Su M. P. Kamikouchi A. (2022). Serotonin modulation in the male *Aedes aegypti* ear influences hearing. Front. Physiology 13, 931567. Available at: https://www.frontiersin.org/articles/10.3389/fphys.2022.931567 . 10.3389/fphys.2022.931567 PMC9465180 36105279

[B107] Yack J. E. (2004). The structure and function of auditory chordotonal organs in insects. Microsc. Res. Tech. 63, 315–337. 10.1002/jemt.20051 15252876

[B108] Yang Z. Yu Y. Zhang V. Tian Y. Qi W. Wang L. (2015). Octopamine mediates starvation-induced hyperactivity in adult *Drosophila* . Proc. Natl. Acad. Sci. 112, 5219–5224. 10.1073/pnas.1417838112 25848004 PMC4413307

[B109] Yorozu S. Wong A. Fischer B. J. Dankert H. Kernan M. J. Kamikouchi A. (2009). Distinct sensory representations of wind and near-field sound in the *Drosophila* brain. Nature 458, 201–205. 10.1038/nature07843 19279637 PMC2755041

[B110] Yu S. Liewald J. F. Shao J. Costa W. S. Gottschalk A. (2021). Synapsin is required for dense core vesicle capture and cAMP-dependent neuropeptide release. J. Neurosci. 41, 4187–4201. 10.1523/JNEUROSCI.2631-20.2021 33820857 PMC8143207

[B111] Yuan Q. Lin F. Zheng X. Sehgal A. (2005). Serotonin modulates circadian entrainment in *Drosophila* . Neuron 47, 115–127. 10.1016/j.neuron.2005.05.027 15996552

[B112] Zhao W. Dewey J. B. Boothalingam S. Dhar S. (2015). Efferent modulation of stimulus frequency otoacoustic emission fine structure. Front. Syst. Neurosci. 9, 168. 10.3389/fnsys.2015.00168 26696843 PMC4674573

[B113] Zhao W. Dhar S. (2011). Fast and slow effects of medial olivocochlear efferent activity in humans. PLoS One 6, e18725. 10.1371/journal.pone.0018725 21494578 PMC3073004

[B114] Zheng J. Shen W. He D. Z. Long K. B. Madison L. D. Dallos P. (2000). Prestin is the motor protein of cochlear outer hair cells. Nature 405, 149–155. 10.1038/35012009 10821263

[B115] Zhukovskaya M. I. Polyanovsky A. D. (2017). Biogenic amines in insect antennae. Front. Syst. Neurosci. 11, 45. 10.3389/fnsys.2017.00045 28701930 PMC5487433

